# An evaluation of the maintenance to abstinence (MTA) program in achieving abstinence in opioid users and improving mental health and quality of life

**DOI:** 10.1186/s13722-019-0132-x

**Published:** 2019-02-04

**Authors:** Madeleine M. Southey, Trent Rees, Margaret Rolfe, Sabrina Pit

**Affiliations:** 1 Western Sydney University, Sydney, NSW Australia; 2 The Buttery, Binna Burra, QLD Australia; 3 University of Sydney, Sydney, NSW Australia; 4 University Centre for Rural Health, 62 Uralba Street, PO Box 3074, Lismore, NSW 2480 Australia; 5 Western Sydney University School of Medicine, Campbelltown, NSW Australia

**Keywords:** Anxiety, Depression, Quality of life, Abstinence, Opioid use, Mental health, Residential rehabilitation, DASS, WHOQOL-8, K10

## Abstract

**Background:**

Residential opioid rehabilitation aims to improve the mental health and quality of life of opioid users through abstinence and residential program participation. This study aimed to determine the depression, anxiety, stress and quality of life amongst maintenance to abstinence (MTA) program residents. Secondary study aims were to assess the personal characteristics of MTA clients, addiction and risk taking behaviours, factors associated with program completion, as well as to assess the reliable change in participants’ mental health and quality of life on exit.

**Methods:**

Retrospective analysis of routinely collected data (2013–2017) from surveys completed by 100 clients. Outcome measures were: Depression, Anxiety, Stress Score (DASS-42), World Health Organisation Quality of Life 8 questions (WHOQOL-8) and Kessler Psychological Distress Scale (K10). Other variables included demographics, drug use, other addictions, aggression, self-harm, suicidal ideation/attempts, and risk taking behaviours. Statistical methods included Chi-square, Fisher’s exact, t-tests, repeated measures analysis of variance and the Reliable Change Index.

**Results:**

All mean DASS-42, WHOQOL-8 and K10 scores improved significantly in all participants from entry to exit (p < 0.001). The majority of participants demonstrated reliable improvement across all psychometric measures. Completion rates for the MTA program were 51%. Depression (p = 0.023), anxiety (p = 0.010) and stress (p = 0.015) DASS-42 scores decreased significantly more in completers compared to non-completers. The rate of improvement in mean WHOQOL-8 scores and psychological distress scores (K10) was not statistically significantly different between completers and non-completers over time. There was no significant difference between completers and non-completers on socio-demographics, self-reported drug addiction or risk taking behaviour on program entry, except for suicidal thoughts while intoxicated (p = 0.033). Completers were more satisfied with their relationships (p = 0.044) and living place (p = 0.040) on program entry.

**Conclusion:**

Overall, completers and non-completers demonstrated improved mental health and quality of life from entry to exit, regardless of program completion. Depression, anxiety and stress reduced more markedly in program completers. Policy makers and programmers could use these findings to further validate their own programs to improve mental health and quality of life of opioid users.

## Background

Residential opioid rehabilitation aims to improve the mental health and quality of life of opioid users through participation in a residential program which includes psychological support. A maintenance to abstinence (MTA) program has the specific goal of achieving abstinence from maintenance therapies, also known as opioid agonist medications, such as Methadone or Buprenorphine. Although the standard of care for treatment of opioid use disorder is with maintenance therapies [1], this program offers individuals who would like to wean off these medications the opportunity to do so in a supportive and structured environment. Alternative residential programs for opioid use disorder include the more traditional Therapeutic Community (TC) program which relies on the effects of the socialisation within a group in a residential setting as the main agent of therapy and change [2]. The challenge that individuals with opioid use disorder may face is that some TC programs require abstinence at entry to the program, or may not be equipped to manage and dispense maintenance therapies for those wishing to be included in a residential program who require maintenance therapies [2, 3]. The MTA program is similar to a TC program in its residential setting and community focus, however differs in that it provides a dedicated service for those individuals who wish to wean off opioid agonist therapies to achieve abstinence.

### The impact of opioid use

Opioid use impacts the individual’s mental health, physical health, social wellbeing and has an impact on Australian society. The negative effects on the individual’s mental health are profound [4] with much higher levels of psychological distress and depression experienced by those who use illicit drugs [5–7] with heroin users experiencing the highest rates of mental illness in this group [8]. Substance use disorder is also the most common major comorbidity in those with severe mental illness [9]. Physical health is also impacted with use of illicit drugs contributing to an estimated 2.6% of Australia’s burden of disease, and 0.5% of deaths in 2010 with major health problems arising from overdose, mental illness, suicide, self-harm, blood borne viruses and death [8]. The self-reported physical health of methadone maintenance patients on entry to a residential rehabilitation program is comparable to that of a person with a severe physical illness [10]. The social impacts include issues of strained family relationships, relationship breakdown and family and intimate partner violence [11]. The impact on Australian society includes increased crime rates, productivity losses and healthcare costs and is estimated to cost the Australian economy $8 billion per year [5, 11, 12].

### Maintenance to abstinence programs

There is an abundance of literature evaluating the utility of different treatment modalities on the mental health and quality of life of opioid users, however there is a gap in the literature for evaluating programs such as the MTA program with a goal of achieving abstinence from maintenance therapies, particularly in Australia.

The UK National Treatment Outcome Research study, a prospective cohort study, studied individuals undergoing treatment for drug addictions in four main treatment modalities (in-patient, residential rehabilitation, methadone reduction and methadone maintenance) [13]. They found that all treatment modalities reduced the frequency of heroin use, non-prescription methadone use, as well as a reduction in injecting and needle sharing in participants over the next 4–5 years of follow up after their stay in a residential rehabilitation program [14]. This study also showed improvements in psychosocial and physical health of participants in any type of treatment for substance use. In a further report of this ongoing study, they found specifically that rates of abstinence were higher in those who attended residential programs and methadone programs. They also found that almost half those who attended the residential rehabilitation treatment were abstinent from heroin 5 years after their treatment [14]. This finding highlights the importance of the residential setting in supporting those with opioid use disorder.

Length of stay has been shown to be a predictor of reliable change in the psychological recovery and well-being of individuals after completion of a residential substance use treatment [15] including participation in a therapeutic community. A therapeutic community is a model of residential rehabilitation for substance use in which a group setting provides therapy for its members by engagement in a community and participation in group psychotherapy [16]. A randomised control trial in Iran that found that involvement in a therapeutic community improved the mental health and quality of life of participants to a greater degree than methadone maintenance or residential rehabilitation, however only if the therapeutic community length of stay (LOS) was greater than 6 months [17]. This study found improvements in psychological wellbeing for those receiving any of the three modalities of treatments offered, being therapeutic communities, methadone maintenance, or residential rehabilitation.

The aforementioned programs, similar to the MTA program, have shown improvements in the quality of life and mental health of participants. The high burden of disease and poor psychometric outcomes for opioid users, as well as the social and economic costs to society highlight that there is great need for programs to address opioid addiction. Moreover, the availability of residential rehabilitation in those with opioid use disorder is of particular importance as this population is more likely to suffer homelessness and social exclusion limiting their access to outpatient services [11]. This study will contribute to the current available literature through evaluating a residential maintenance to abstinence program in an Australian setting.

This study aimed to primarily determine the depression, anxiety, stress and quality of life levels amongst MTA program residents at program entry and exit. Further aims of this study were to assess the personal characteristics of individuals participating in the Maintenance to Abstinence (MTA) programs and to identify which factors on entry were associated with MTA program completion. The study also aimed to assess the changes in psychometric parameters over time, and evaluate the reliability of the change in these measures between entry and exit of the program.

## Method

This study was a retrospective analysis of routinely collected data from all individuals aged 18 years and over who attended the MTA program between 2013 and 2017 and consented to participating in research evaluation. All participants of the MTA program who consented for this data to be used for research purposes were included in this study.

### Research in context

The maintenance to abstinence program (MTA) is a residential rehabilitation program for individuals with opioid dependence and misuse who aim to achieve abstinence. Entry to the program requires an opioid use history of over 2 years and a personal desire to achieve abstinence, not driven by a condition of bail or other contributing legal pressures.

Individuals attend the program voluntarily and are assisted in reducing, and then achieving abstinence from their maintenance therapy (either Buprenorphine or Methadone) during their stay. Their maintenance medication is then reduced as per the NSW clinical guidelines for the treatment of opioid dependence [18], however they will also meet with a prescriber once per week to review their withdrawal severity, and dose reductions are therefore managed for the individual case.

The program also incorporates psychological support such as counselling and stress management exercises, as well as the opportunity for social engagement through becoming involved in activities, education and low intensity work on site. The aims of these practices are to build self-esteem and reduce mental illness in the residents throughout their rehabilitation. The program also supports the physical health of the residents through regular healthy meal times and encouragement to exercise. The program includes guidance and counselling to teach strategies to cope and remain abstinent after leaving the program [19]. These importance of these psychosocial aspects of the program also follow the NSW clinical guidelines for opioid dependence for those who are achieving abstinence from their maintenance therapies [18].

Participants may leave voluntarily at any point throughout the program, as well as at completion of the program. Upon achieving abstinence, participants may also be given the choice to progress into a therapeutic community program in the same facility. Participants engage with a group and are responsible for domestic tasks, and participate in leisure activities with support from therapists and psychologists. Participants may be asked to leave the program if they are unable to follow the restrictions of substance use in the program, as well as general social and safety requirements of the facility. These participants are considered to have left the program “involuntarily” [19].

### Participants and data collection

Data was routinely collected by staff and through computer based questionnaires. Data was collected on entry to the program, at the halfway point (6 weeks) and on exit of the program (12 weeks, or at the point at which a participant left the program). Participants provided written consent for this data to be used for research purposes. De-identified data was provided to the research team.

Participants were considered to have completed the program if they completed the 91 days in the MTA program, or had achieved abstinence early and progressed to the Therapeutic Community program at the same location.

Ethics approval was given by the University of Western Sydney Human Research Ethics Committee (EC00314), approval number H11353.

## Outcome measures

The primary outcomes measures were quality of life and mental health status of the participants. This involved the use of the psychometric tools the Depression, Anxiety, Stress Scale (DASS) [20], the World Health Organisation Quality of Life scale (WHOQOL-8) [21] and the Kessler Psychological Distress Scale (K10) [22].

The DASS-42 scale measures levels of depression, anxiety and stress as separate subscales Each subscale has a minimum score of zero, maximum score of 42. A depression score of 0–9 indicates normal levels of depressive symptoms, a score of 10–13 mild, 14–20 moderate, 21–27 severe and over 28 extremely severe depression. An anxiety score of 0–7 indicates normal anxiety levels, 8–9 mild, 10–14 moderate, 15–19 severe and over 20 extremely severe anxiety. A stress score of 0–14 indicates normal stress levels, 15–18 mild, 19–25 moderate, 26–33 severe and over 34 extremely severe stress [23].

WHOQOL-8 is validated for use in Australia [24]. It is a condensed version of the WHOQOL-100 which is specifically adapted for substance use and mental health disorders. It asks questions in eight domains of quality of life with a scoring system of 1–5, where high scores infer higher satisfaction. The total score of the WHOQOL-8 is the summation of the scores in all eight domains giving a total score of 8–40. The domains are health, energy, money, daily living, self-satisfaction, relationships, living place, and quality of life which reflects the individual’s overall perception of quality of life. The score of the WHOQOL-8 reflects an individual’s perceived quality of life and was used to assess any change in perceived quality of life over the course of the MTA program.

The Kessler Psychological Distress Score (K10) measures levels of psychological distress. A high K10 score correlates to high levels of psychological distress, and may indicate an increased likelihood of depression or an anxiety disorder. A score under 20 indicates low levels of psychological distress, a score from 20–24 mild levels, 25–29 moderate levels, and over 30 severe levels of psychological distress [25]. The minimum score of the K10 is 10, and the maximum 50. These scores were used to measure mental health at various stages of the MTA program.

The Modified Monash Model (MMM) [26] is a tool used to describe degrees of rurality and remoteness across Australia. It has five categories ranging from MMM1 (Modified Monash Model level 1) indicating an urban centre, with increasing levels of rurality and isolation from an urban centre. MMM5 (Modified Monash Model level 5) refers to regional areas which are not within 10 km of a town with a population of 5000–15,000 people.

Further variables that were collected were the participants’ age, gender, primary substance of use, age at first use, other addictions, aggression, self-harm and suicidal ideation/attempts, risk taking behaviour and gambling problems.

## Statistical analyses

Descriptive statistics were used to determine the demographics of the cohort, as well as the means of the various outcome measures. Chi-square, Fisher’s exact, and t-tests were used to determine the characteristics associated with program completers versus non-completers. A *p* value of less than 0.05 was considered to be significant. Repeated measures analysis of variance (RMAV), using SPSS version 24 [27], was used to assess the psychometric measurements (individual DASS-42 and WHOQOL-8 scores and total K10 scores) over time (entry compared to exit) where the time by group (completer/non-completer) interaction indicates a difference in the rate of change between completers and non-completers over time.

The Reliable Change Index was calculated for the total K10, WHOQOL-8 and DASS-42 scores in line with Turner and Deane [15] and was based on the Christensen and Mendoza formula [28]. It evaluates significant individual change. Participants individual exit and entry scores were subtracted and then divided by the standard error of the difference according to the formula:

$$ RC = \frac{{x_{2} - x_{1} }}{{S_{diff} }} $$

where $RC$ = relative change, $x_{2}$ = exit scores, $x_{1}$ = entry scores, $S_{diff}$ = standard error of the difference.

A change score for an individual was considered significant if it was outside the two standard deviations. Participants within the two standard deviations were classified as ‘reliably not improved’. Participants on either side of the normal curve of two standard deviations were classified as either ‘reliably improved’ or ‘reliably declined’. Each category of reliable change was compared with length of stay in the program. Given the small numbers and the non-normal distribution of length of stay, it was not possible to compare the mean length of stay with each reliable change category. Thus, length of stay was divided into three categories: less than 46 days (1st quartile), 47–82 days (second quartile) and more than 83 days. Following this, a Fisher’s exact test was used to determine whether there was an association between reliable change categories and length of stay.

## Results

The inclusion criteria was met by 100 individuals, 14 individuals did not consent to their data being used for research purposes and were therefore excluded from this study.

### Completion of program

Table 1 shows that 51% of participants completed the program. Of the completers, 23% completed the full program, and 28% progressed to the therapeutic community. Fifteen percent left the program voluntarily without completing (Table 1). A minority of participants left the program involuntarily (15%), 19% left voluntarily, and 9% left involuntarily after reducing their maintenance opioid dose. Only 6% of participants left involuntarily without achieving a reduction in their maintenance opioid dose.

**Table 1:** Completion, length of stay, demographic differences and primary drug addiction behaviours in completers versus non-completers

	Total		Completers	Non-completers	p value
	N = 100		N = 51	N = 49	
	n	%	%	%	
Exit status					< 0.001^3^
Voluntary	85	85	51	34	
Program complete	23	23	23	0	
Program incomplete	15	15	0	15	
Progression to therapeutic community	28	28	28	0	
Reduction complete	19	19	0	19	
Involuntary	15	15	0	15	
Reduction complete	9	9	0	9	
Reduction incomplete	6	6	0	6	
Length of stay (LOS) in days^a^					< 0.001^b^
< 47	25	25	0	25	
47–82	23	23	3	20	
> 82	52	52	48	4	
Gender					
Male	56	56	53.1	58.8	0.562^b^
Female	44	44	46.9	41.2	
Age (years)					
< 30	9	9	5.9	12.2	
30–39	43	43	39.2	46.9	
40–49	37	37	56.8	32.7	
> 50	11	11	13.7	8.2	
Mean age in years (sd)	38.9	(7.7)	40.1 (7.7)	37.6 (7.5)	0.094^c^
Rurality (n = 98)					0.901^c^
MMM1	63	62	64.7	63.8	
MMM2	3	3	2.0	4.3	
MMM3	9	8	7.8	10.6	
MMM4	15	15	17.6	12.8	
MMM5	8	8	7.8	8.5	
Mean MMM score (sd)	2.0	(1.5)	2.0 (1.5)	2.0 (1.4)	0.890^c^
Primary substance					0.781^b^
Analgesics	80	80	82.4	77.6	
Sedatives and hypnotics	12	12	9.8	14.3	
Stimulants and antipsychotics	8	8	7.8	8.2	
Method of use					0.773^b^
Inject	65	65	70.6	59.2	
Ingest	25	25	19.6	30.6	
Absorption	2	2	2.0	2.0	
Smoke	4	4	3.9	4.1	
Sniff (powder)	1	1	2.0	0.0	
Other	3	3	2.0	2.0	
Last injected (n = 99)					0.614^b^
Within previous 3 months	65	65	66.7	63.3	
More than 3, less than 12 months	12	12	13.7	10.2	
More than 12 months ago	11	11	11.8	10.2	
Never Injected	12	12	7.8	16.3	

*sd* standard deviation, *MMM* Modified Monash Model

^a^A completer stay of less than 91 days indicates an early attainment of abstinence and progression to the therapeutic communities program at the same facility

^b^p value for Chi-squared test

^c^p value for t-test

### Population sample and drug use behaviour

The sample population is comprised of 56% males and 44% females. The mean age is 38.9 (SD = 7.7) years, with 80% of participants aged between 30 and 49 years. The majority of participants (62.4%) were from a major city [26]. There was no significant difference between completers and non-completers for gender, age, rurality, primary drug addiction behaviour, method of use or last injection (Table 1).

Twelve percent of participants on entry reported non-drug addictions (sex, TV, food, video games, gambling, internet, shopping or exercise), one third reported a problem with aggression and about half reported problems with self-harm or suicidality and 59% reported risk taking behaviour more than once a month. The only statistically different behaviour between completers and non-completers reported on entry was that a significantly higher number of completers experienced suicidal thoughts while intoxicated (p = 0.033). Most variables had very small numbers (Table 2).

**Table 2:** Self-reported problems in completers versus non-completers on entry

	Total		Completers	Non-completers	p value
	N = 100		N = 51	N = 49	
	n	%	%	%	
General groupings of self-reported problems					
Any non-drug addictions	12	12.0	11.8	12.2	0.941
Aggression	33	33.0	31.4	34.7	0.724
Self-harm/suicidality	52	52.0	47.1	57.1	0.313
Risk taking behaviours more than monthly	59	59.0	62.7	55.1	0.437
Recent self harm and suicidality (in the last 4 weeks)					
Thoughts of self-harm	40	39.6	33.3	46.9	0.165
Thoughts of self-harm while intoxicated	8	7.9	9.8	6.3	0.716
Suicidal thoughts	42	41.6	39.2	44.9	0.565
Suicidal thoughts while intoxicated	11	10.9	17.6	4.2	0.033
Self harm attempts	18	17.8	15.7	20.4	0.539
Self harm attempts while intoxicated	3	3.0	2.0	4.2	0.610
Suicide attempts	19	18.8	18.0	21.3	0.684
Suicide attempts while intoxicated	5	5.0	5.9	4.2	1.000
Current self harm and suicidality on entry					
Current self harm behaviour	1	1.0	0.0	2.0	0.490
Current suicide attempts	1	1.0	0.0	2.0	0.490
Antisocial behaviour					
Abusive language	32	31.7	31.4	32.7	0.891
Abusive language while intoxicated	5	5.0	3.9	6.3	0.672
Aggressive behaviour	8	7.9	5.9	10.2	0.483
Aggressive behaviour while intoxicated	2	2.0	0.0	4.1	0.238
Disciplinary action due to aggression behaviour	1	1.0	2.0	0.0	1.00
Frequency of sharing needles					
Monthly or more	9	9.0	11.8	10.2	0.574
Less than monthly	17	17.0	21.6	12.2	
Once	25	25.0	21.6	28.6	
Never	49	49.0	47.1	51.0	
Frequency of unsafe sex (n = 100)					
Never	72	72.0	64.7	79.6	0.097
Less than monthly/monthly/weekly or more	28	28.0	35.3	20.4	

p value for Chi-squared test

### Mental health and quality of life in participants

Table 3 shows that on entry the majority of participants showed moderate to very severe depression (76%), anxiety (78%) and stress (63%). K10 scores showed some missing data throughout, which should be taken into account when interpreting the data. There were no statistically significant differences between completers and non-completers in DASS-42 or K10 entry scores.

**Table 3:** Mental health entry scores in completers versus non-completers

	Total		Completers	Non completers	p value^a^
	N = 100		N = 51	N = 49	
	n	%	%	%	
Depression on entry (DASS)					0.910
Normal–mild	24	24.0	23.5	24.5	
Moderate–very severe	76	76.0	76.5	75.5	
Anxiety on entry (DASS)					0.390
Normal–mild	22	22.0	25.5	18.4	
Moderate–very severe	78	78.0	74.5	81.6	
Stress on entry (DASS)					0.718
Normal–mild	37	37.0	35.3	38.8	
Moderate–very severe	63	63.0	64.7	61.2	
Level of psychological distress (K10) on entry^b^					0.372
None	13	13.5	14.3	12.8	
Mild	16	16.7	18.4	14.9	
Moderate	20	20.8	26.5	14.9	
Severe	47	49.0	40.8	57.4	

*DASS-42* Depression, Anxiety, Stress Scale, *K10* Kessler 10

^a^p value for Chi-squared test

^b^5 missing

WHOQOL-8 showed only 21% of participants reporting a “good” quality of life on entry to the program whilst 48% reported “poor” or “very poor” quality of life (Table 4). Participants scored considerably lower on self-satisfaction with 72% reporting being either “dissatisfied” or “very dissatisfied”. Most quality of life domains on entry showed no significant difference between completers and non-completers, except for living place and relationships. Those who completed the program were less dissatisfied with their relationships with 49% of completers compared to 65% of non-completers being either “dissatisfied” or “very dissatisfied” on program entry (p = 0.044). Thirty seven percent of completers were either “dissatisfied” or “very dissatisfied” with living place compared to 45% of non-completers on program entry (p = 0.040).

**Table 4:** Quality of life as measured by WHOQOL-8 on entry in completers versus non-completers

	Total		Completers	Non-completers	p value^a^
	N = 100		N = 51	N = 49	
	n	%	%	%	
*WHOQOL-8 on entry*					
Overall perception of quality of life					
Very poor	12	12	5.9	18.4	0.131
Poor	36	36	35.3	36.7	
Neither poor nor good	31	31	39.2	22.4	
Good/very good	21	21	19.6	22.4	
Overall perception of health					
Very dissatisfied	24	24	17.6	30.6	0.363
Dissatisfied	40	40	43.1	36.7	
Neither satisfied nor dissatisfied	24	24	23.5	24.5	
Satisfied/very satisfied	12.0	12	15.7	8.2	
Enough energy for daily life					
Not at all	21	21	21.6	20.4	0.422
A little	32	32	25.5	38.8	
Moderately	29	29	35.3	22.4	
Mostly	17	17	15.7	18.4	
Completely	1	1	2.0	0.0	
Enough money for daily needs					
Not at all	20	20	15.7	24.5	0.260
A little	37	37	39.2	34.7	
Moderately	17	17	23.5	10.2	
Mostly	21	21	15.7	26.5	
Completely	5	5	5.9	4.1	
Daily living					
Very dissatisfied	21	21	15.7	26.5	0.251
Dissatisfied	32	32	27.5	36.7	
Neither satisfied nor dissatisfied	20	20	21.6	18.4	
Satisfied	23	23	31.4	14.3	
Very satisfied	4	4	3.9	4.1	
Self-satisfaction					
Very dissatisfied	25	25	23.5	26.5	0.556
Dissatisfied	47	47	43.1	51.0	
Nether satisfied nor dissatisfied	16	16	19.6	12.2	
Satisfied	11	11	13.7	8.2	
Very satisfied	1	1	0.0	2.0	
Relationships					
Very dissatisfied	21	21	11.8	30.6	0.044
Dissatisfied	36	36	37.3	34.7	
Neither satisfied nor dissatisfied	24	24	29.4	18.4	
Satisfied	14	14	19.6	8.2	
Very satisfied	5	5	2.0	8.2	
Living place					
Very dissatisfied	18	18	11.8	24.5	0.040
Dissatisfied	23	23	25.5	20.4	
Neither satisfied nor dissatisfied	22	22	25.5	18.4	
Satisfied	26	26	33.3	18.4	
Very satisfied	11	11	3.9	18.4	

*WHOQOL-8* World Health Organisation Quality of Life 8 Questions

^a^p value for Chi-squared test

Table 5 compares completers and non-completers (group) to their change in DASS, WHOQOL-8 and K10 from entry to exit (time). From the repeated measures ANOVA, all psychometric measures improved significantly over time (entry to exit) for both groups with p < 0.001 for all measures. For the time by completion interaction effect, only the three DASS-42 subscales demonstrated significance; depression (p = 0.023), anxiety (p = 0.010) and stress (p = 0.015). This indicates a significantly faster rate of change between entry and exit for completers compared to non-completers over the duration of the program.

**Table 5:** Comparison between completers and non-completers (group) on change in DASS, WHOQOL-8 and K10 from entry to exit (time)

	Completers		Non-completers		Time effect			Completion effect			Time * completion effect		
	Entry	Exit	Entry	Exit	DF	F	P	DF	F	P	DF	F	P
	Mean (SD)	Mean (SD)	Mean (SD)	Mean (SD)									
DASS													
Depression	20.5 (8.61)	7.8 (6.30)	20.2 (10.01)	12.8 (10.42)	(1,90)	82.37	< 0.001	(1,90)	2.51	0.117	(1,90)	5.35	*0.023*
Anxiety	16.5 (8.97)	6.4 (5.49)	16.9 (10.77)	12.3 (9.94)	(1,90)	51.64	< 0.001	(1,90)	4.05	*0.047*	(1,90)	7.02	*0.010*
Stress	20.8 (7.64)	10.2 (6.91)	21.5 (9.23)	16.2 (9.92)	(1,90)	54.66	< 0.001	(1,90)	5.81	*0.018*	(1,90)	6.12	*0.015*
WHOQOL-8													
Total score	20.8 (5.68)	29.3 (5.69)	19.2 (6.67)	24.9 (8.57)	(1,91)	80.42	< 0.001	(1.91)	6.88	*0.010*	(1,91)	3.27	0.074
Quality	1.7 (0.85)	2.9 (0.84)	1.6 (1.03)	2.3 (1.16)	(1,90)	55.87	< 0.001	(1.90)	5.96	*0.017*	(1,90)	3.25	0.075
Health	1.4 (0.96)	2.6 (0.92)	1.1 (0.95)	2.1 (1.15)	(1,90)	73.58	< 0.001	(1,90)	5.35	*0.023*	(1,90)	1.10	0.298
Energy	1.5 (1.07)	2.4 (0.94)	1.4 (1.05)	1.9 (1.26)	(1,90)	30.49	< 0.001	(1,90)	3.08	0.083	(1,90)	2.49	0.118
Money	1.6 (1.12)	2.5 (1.15)	1.5 (1.21)	2.0 (1.33)	(1,90)	26.15	< 0.001	(1,90)	1.47	0.229	(1,90)	1.55	0.216
Daily living	1.8 (1.17)	2.7 (0.92)	1.4 (1.14)	2.3 (1.14)	(1,90)	37.06	< 0.001	(1,90)	5.84	*0.018*	(1,90)	0.01	0.935
Self-satisfaction	1.2 (0.97)	2.6 (0.98)	1.1 (0.87)	2.1 (1.28)	(1,90)	81.20	< 0.001	(1,90)	3.26	0.074	(1,90)	2.32	0.131
Relationships	1.6 (1.00)	2.6 (0.90)	1.3 (1.21)	2.2 (1.17)	(1,90)	52.19	< 0.001	(1,90)	3.94	*0.050*	(1,90)	0.16	0.692
Living place	1.9 (1.11)	3.0 (0.79)	1.8 (1.50)	2.6 (1.22)	(1,90)	39.33	< 0.001	(1,90)	1.71	0.194	(1,90)	1.12	0.292
K10^a^													
Total score	28.1 (8.00)	19.3 (5.91)	31.0 (8.43)	23.7 (8.84)	(1,70)	44.30	< 0.001	(1,70)	6.03	*0.017*	(1,70)	0.41	0.525

Significant data points are highlighted using italic typeface (significant P values)

*DF* degrees of freedom (variance), *F* F ratio, *P* p value for F test

^a^K10 date is missing 5 responses from entry and 28 responses from exit

The mean reported depression score at entry is “moderate depression” for completers (20.5) and non-completers (20.2) when scored on the DASS-42 scoring system, the mean on exit is “normal” (7.8) for completers and “mild depression” for non-completers (12.8). Anxiety on entry was “severe” for completers (16.5) and non-completers (16.9), which dropped to “normal” for completers (6.4) and “moderate” for non-completers (12.3) on exit. Stress levels on entry were “moderate” for both completers (20.8) and non-completers (21.5), dropping to “normal” for completers (10.2) and “mild” for non-completers (16.2) on exit. Depression (p = 0.023), anxiety (p = 0.010) and stress (0.015) scores decreased significantly more for those that completed the MTA program overtime compared to those who did not. Only anxiety (p = 0.047) and stress (p = 0.018) had a significant group (completion) effect indicating that these scores averaged over time were significantly greater in the non-completion group. There was no statistically significant difference on depression, anxiety and stress on entry between completers and non-completers (results not shown).

Completers had higher mean scores for all WHOQOL-8 scores when compared to non-completers on entry and exit. All mean WHOQOL-8 scores improved significantly over time from entry to exit, all with a p value of < 0.001 but the rate of improvements were not statistically significantly different between completers and non-completers over time. Only the total quality of life score (p = 0.010) quality of life (p = 0.017), health (p = 0.023), daily living (p = 0.018) and relationships (0.050) had a significant group (completion) effect indicating that these domains averaged over time were significantly higher in the completion group. There was no statistically significant difference on WHOQOL-8 scores on entry between completers and non-completers, with the exception of daily living (p = 0.042) (results not shown).

Mean K10 scores on entry correlated with “moderate levels of psychological distress” (28.1) for completers and “severe” for non-completers (31.0), which dropped to “none” for completers (19.3) and “mild” for non-completers (23.7) on exit. The K10 score improved significantly over time from entry to exit (p < 0.001) but the rate of improvements were not statistically significantly different between completers and non-completers over time (p = 0.525). K10 scores had a significant group (completion) effect (p = 0.017) indicating that these domains averaged over time were significantly greater in the non-completion group.

There was no statistically significant difference for psychological distress on entry between completers and non-completers.

Table 6 shows that the majority of participants reliably improved on all scores. Length of stay and reliable change scores for the K10, WHOQOL-8 and DASS-42 were not statistically different between the groups. DASS-42 scores improved for 72.8% of participants, WHOQOL-8 scores reliably improved for 76.3% of participants, and K10 reliably improved in 74.0% of participants. The proportion of those individuals that reliably improved was the highest in the longest length of stay (83 days or more) group with 80.8%, 78.8% and 79.6% reliably improved in the DASS-42, WHOQOL-8 and K10 scores respectively. There was no statistical significance between and increased length of stay and reliable improvement.

**Table 6:** Proportion of participants classified as reliably improved, not improved or declined on K10, total WHOQOL-8 and total DASS-42 scores and differences in length of stay (days) between groups (N = 93)

Total sample				
Measure	% Reliably improved (n)	% Not improved (n)	% Reliably declined (n)	p value
DASS-42 (N = 92)	72.8 (67)	19.6(18)	7.6 (7)	
WHOQOL-8 (N = 93)	76.3 (71)	12.9 (12)	10.8(10)	
K10 (N = 73)	74.0 (54)	13.7 (10)	12.3 (9)	
Length of stay (days)				
DASS-42 (N = 92)				
≤ 46 days	71.4 (15)	23.8(5)	4.8 (1)	0.088
47–82	52.6 (10)	26.3 (5)	21.1(4)	
83+	80.8 (42)	15.4 (8)	3.8 (2)	
WHOQOL-8 (N = 93)				
≤ 46 days	72.7 (16)	13.6 (3)	13.6 (3)	0.937
47–82	73.7 (14)	15.8 (3)	10.5 (2)	
83+	78.8 (41)	11.5 (6)	9.6 (5)	
K10 Scores (N = 73)				
≤ 46 days	58.3 (7)	33.3 (4)	8.3 (1)	0.158
47–82	66.7 (8)	8.3 (1)	25 (3)	
83+	79.6 (39)	10.2 (5)	10.2 (5)	

p value for Fisher’s Exact Test for comparisons between groups on length of stay in days

## Discussion

### Statement of principle findings

The MTA program appears to improve the mental health and quality of life of those with opioid addiction through involvement in the program, regardless of whether or not they complete the program. Only depression, anxiety and stress reduced more markedly in program completers. Quality of life and psychological distress improved for both completers and non-completers but this improvement was not significantly higher among completers. Reliable improvement was shown across all parameters for the majority of participants, with 74% showing reliable improvement in K10 score, 76.3% showing reliable improvement in WHOQOL-8 score and 72.8% showing reliable improvement in overall DASS-42 score. This data strengthens the notion that an MTA program is effective in improving mental health and quality of life in opioid users. Factors measured on entry that were associated with completion were suicidal thoughts while intoxicated and the participant’s satisfaction with their relationships and living place. This information could be used to develop strategies around improving these measures, or monitoring those who scored lower in these measures which are associated with non-completion.

### Meaning of the study

Improvements in mental health and quality of life should be expected when an individual is supported to remain abstinent from opioid drugs, as the relationship between the use of these substances and poor mental health and quality of life measurements is clearly documented [4, 5, 9]. The results of this study support this body of evidence as improvements in mental health and quality of life parameters were seen among the majority of participants in the program to some degree regardless of completing the program in its entirety. However, completers had significantly greater improvements in their depression, anxiety and stress scores than non-completers. This shows that the program when followed through to completion is associated with better mental health outcomes than partial participation in the program. The quality of life domains improved for both groups but the improvements were not higher in the completion group. It is possible that the individual quality of life domains measured through one question only are not strong enough indicators on their own. The total WHOQOL-8 score was trending towards significance (p = 0.077). The fact that the K10 scores were not significantly different between completers and non-completers may be a reflection of the high amount of missing data in this outcome measure (16%).

An interesting finding of the study was the association between completion, and less dissatisfaction with living place and relationships according to the WHOQOL-8 score *on entry* to the program when comparing the level of satisfaction. Living place and relationships have been described as “fundamental human needs” since psychologist Maslow first coined the phrase in 1943 [29]. Living place is of particular concern when supporting those recovering from opioid addiction as there is increased prevalence of unstable housing and homelessness in this population [11, 30]. This may be another reason that residential settings have been shown to be effective in this population, and important in considering the availability of residential options for opioid use disorder treatment. The fact that only these two quality of life domains measured on entry into the program were associated with completion rates may indicate the importance of living place and relationships in promoting recovery in opioid users. This supports the notion that opioid rehabilitation needs to include social support as well as the involvement of social workers to address the needs of individuals, including practical needs around their living place. It is important to note that due to multiple comparisons the findings can be type 1 error findings and thus maybe due to chance. Additionally, our sample size was relatively small which may have limited our ability to find statistically significant differences between completers and non-completers. It may therefore be useful to re-evaluate these factors when the facilities client base has grown over time.

Another statistically significant association with completers was higher incidence of suicidal thoughts while intoxicated in the 4 weeks before entry to the program. No other self-harm, suicidality while sober, or other risk taking behaviour was associated with completion. It may be of interest to look at an association with levels of alcohol consumption between the two groups to see if this result may be explained by levels of alcohol intoxication. This finding may be due to chance considering that there were no other associations found.

Quality of life and mental health significantly improved over time (entry to exit) for both completers and non-completers (Table 5). This demonstrates that the program has significant benefit even without continuing to completion. It may be that the opportunity and exposure to psychological therapies in the program leads to improvements in mental health and quality of life even with an earlier exit time. The psychological support offered in the MTA program is evidence based in improving mental health outcomes in individuals suffering substance use disorders [31–33]. The fact that the rate of improvement was better in the completers group when looking at the time by completion interaction effect may indicate that the exposure to psychological interventions for the entirety of the program supports a better rate of improvement in depression, anxiety and stress. This is an important finding for a residential rehabilitation program as it validates their methods throughout the program, showing improved outcomes even amongst those that do not finish the program. There was no significantly different improvement rates in completers K10 scores when looking at the time, completion interaction. Given that K10 is also a measure of mental health outcomes this is a surprising finding considering the significantly better rates of improvement in the DASS-42 parameters for completers. An explanation for this may be that the missing data in K10 exit scores gave too small a sample size for a true analysis of rate of improvement in psychological distress.

Reliable improvement was seen across all outcome measures for the majority of participants, strengthening the conclusions of this study. Individuals who remained in the program for more than 83 days had a higher proportion of individuals who showed reliable improvement across all measures. The majority of people who stayed less than 46 days also showed reliable improvements. Increased length of stay was not found to be a statistically significant indicator of improved mental health and quality of life outcomes for participants. Those with the longest length of stay (more than 83 days) having the highest proportion of reliable improvement across all measures may give some support to the current literature which indicates that increased length of stay, particularly of 90 days or more, relates to higher rates of reliable improvement [15], however, the association was not significant in our study. With increased numbers of participants finishing the program in the future it may be of value to repeat this analysis with a larger sample size.

### Limitations and strengths

Study limitations were the small sample size, self-reported data and relatively high missing data for some variables, especially the K10 data. The MTA program data was only available over a four year period, which led to the small sample size for this study and may therefore have been underpowered to detect differences between completers and non-completers. Another limitation is that this study does not draw comparison to other treatment modalities such as individuals remaining on maintenance therapies, nor those in a therapeutic community program. Furthermore, the study took place in one particular setting so may not be generalisable to other settings.

### Relation to other studies

The findings of this study support the body of literature which discusses the utility of residential rehabilitation for opioid users on improving their psychosocial wellbeing [14, 15]. The association between increased mean length of stay in residential substance use treatment, and reliable improvement over all outcome measures supports the findings of Turner and Deane [15].

A study was conducted on the Therapeutic Community program at the same location [34]. Completion rates for this program were lower, however the program itself is significantly longer (217 days as opposed to 98). The DASS-42 and WHOQOL-8 scores for participants in this study were however similarly significantly improved. This results of this study with regard to predictors of completion differed, and included age and high WHOQOL-8 scores in the domain of money, which were not found to be significant in this study. It is noted that the sample size for that study was larger (n = 257).

### Future research

Future research for both the Buttery’s MTA program, as well as other residential opioid rehabilitation programs, would be to conduct long term follow up studies. It would be of interest to examine the same outcome measures of mental health and quality of life at intervals after exit from the program. A survey detailing relapse of opioid or other drug use would be of interest to determine whether abstinence is maintained long term in those who completed the program. Further studies of MTA programs could aim to compare the characteristics of individuals with different dose reduction regimens, as well as withdrawal effect profiles.

### Impact of research outcomes

This study adds to the evidence supporting the use of MTA programs such as The Buttery’s. It validates residential MTA rehabilitation as a means of intervention for those affected by opioid use, even if they do not complete the full program. Another potential impact from this study may be that MTA programs continue to improve the mental health and quality of life outcomes for those suffering from opioid use. This may subsequently reduce the impact that these individuals may have on other health services later.

This study may assist rehabilitation program providers to further improve their understanding of the needs of individuals throughout their recovery. In particular, it points to the important and basic needs of individuals to have satisfactory relationships and living arrangements in order to best support their recovery. This may give them a greater chance of program completion. This finding could be used to help identify individuals who are at risk of program drop out, and potentially lower their risk by focussing on improving their satisfaction with both their living place and relationships.

The MTA program in particular are incorporating the findings of this study into practice. Specifically they are reviewing the need for flexibility in length of stay, as well as developing a profile of the significant associations with completion, in order to reflect on how better to meet the needs of the client group who exit the program early.

In conclusion, the use of the MTA program shows reliable improvements in opioid users’ mental health and quality of life, irrespective of program completion. Depression, anxiety and stress show increased rates of improvements amongst those that complete the program. These findings may help guide decisions about service requirements for people who are recovering from drug misuse and support the continued use of residential opioid rehabilitation programs.

## Appendices

### MMM Modified Monash Model

- MMM1: Major cities.
- MMM2: Regional but within 20 km of a town with a population over 50,000.
- MMM3: Regional that are within 15 km road distance to a town of population 15,000–50,000.
- MMM4: Regional and are in 10 km road distance from a town with a population of between 5000 and 15,000.
- MMM5: Regional and not within 10 km distance of a town of population 5000–15,000.

Table legend:

DASS-42 categorical legend [21]:

**Table:**

	Normal	Mild	Moderate	Severe	Very severe
Depression	0–9	10–13	14–20	21–27	28–42
Anxiety	0–7	8–9	10–14	15–19	20–42
Stress	0–14	15–18	15–25	26–33	34–42

K10 categorical legend [22]:

**Table:**

K10 Score (total)	Level of psychological distress
10–19	The score indicates that the client or patient may currently not be experiencing significant feelings of distress
20–24	The client or patient may be experiencing mild levels of distress consistent with a diagnosis of a mild depression and/or anxiety disorder
30–50	The client or patient may be experiencing severe levels of distress consistent with a diagnosis of a severe depression and/or anxiety disorder

## Abbreviations

ANOVA: analyses of variance
DASS-42: Depression, Anxiety, Stress Score
K10: Kessler Psychological Distress Scale
LOS: length of stay
MMM: Modified Monash Model
MTA: maintenance to abstinence
STD: standard deviation
WHOQOL-8: World Health Organisation Quality of Life 8 questions
